# Description d’un nouveau foyer épidémique de leishmaniose cutanée à *Leishmania major* à l’Ouest du Burkina Faso

**DOI:** 10.11604/pamj.2020.35.65.20825

**Published:** 2020-03-06

**Authors:** Issouf Konate, Ibrahim Sangare, Jacques Zoungrana, Ziemlé Clément Meda, Christophe Kafando, Yacouba Sawadogo, Rock Dabiré, Nicolas Meda, Boukary Diallo, Jean-Baptiste Andonaba, Fatou Barro-Traoré, Pascal Niamba, Adama Traoré

**Affiliations:** 1 Institut Supérieur des Sciences de la Santé, Université Nazi Boni, Bobo-Dioulasso, Burkina Faso; 2 Centre Muraz, Institut National de Santé Publique, Bobo-Dioulasso, Burkina Faso; 3 Direction de la Protection de la Santé, Ministère de la Santé, Ouagadougou, Burkina Faso; 4 Direction Régionale de la Santé des Hauts Bassins, Bobo-Dioulasso, Burkina Faso; 5 Institut de Recherche en Sciences de la Santé, Direction Régionale de l’Ouest, Bobo-Dioulasso, Burkina Faso; 6 Unité de Formation et de Recherche en Sciences de la Santé, Université Joseph Ki-Zerbo, Ouagadougou, Burkina Faso

**Keywords:** Cutaneous leishmaniasis, epidemic, Leishmania major, Burkina Faso, Leishmaniose cutanée, épidémie, *Leishmania major*, Burkina Faso

## Abstract

**Introduction:**

au Burkina Faso, le seul foyer de leishmaniose cutanée (LC) avec confirmation biologique dans la littérature à notre connaissance est celui de Ouagadougou au centre. Nous rapportons les résultats épidémiologiques, cliniques et biologiques de l’investigation d’un nouveau foyer épidémique à Larama, à l’ouest du Burkina.

**Méthodes:**

la méthode du camp a été utilisée pour recevoir les cas. Les données sociodémographiques et cliniques ont été recueillies à l’aide d’un questionnaire. La confirmation a été faite par la microscopie puis par réaction en chaîne par polymérase (PCR).

**Résultats:**

au total, 108 cas suspects ont été recensés à Larama, soit un taux d’attaque de 5,8%. Le sex ratio était de 1,08. Les patients concernés étaient le plus souvent des cultivateurs (35,2%) et des commerçants (33,3%). La population active (15 à 49 ans) représentait 51,9%. Le nombre de lésion variait entre 1 et 5 dans 91,7% des cas. Les lésions qui se présentaient sous formes ulcéro-crouteuses surélevées et infiltrées, étaient localisées sur les membres (87%) avec une durée d’évolution entre un et cinq mois dans 96,3% des cas. Sur les deux cas prélevés, la microscopie a montré des leishmanies et la PCR a confirmé l’espèce *Leishmania major*.

**Conclusion:**

nos résultats confirment l’existence d’une épidémie de leishmaniose cutanée à *L. major* dans l’ouest du pays. Des enquêtes complémentaires sont nécessaires pour préciser la charge de morbidité des leishmanioses au Burkina Faso.

## Abstract (en)

**Introduction:**

in Burkina Faso, the only epidemic focus of cutaneous leishmaniasis confirmed in the literature by lab tests was in Ouagadougou. We report the epidemiological, clinical and biological results of the assessment of a new epidemic focus in Larama in western Burkina Faso.

**Methods:**

camps were used to receive patients. Sociodemographic and clinical data were collected using a questionnaire. Confirmation was based on microscopy and polymerase chain reaction (PCR).

**Results:**

a total of 108 suspected cases have been identified in Larama, reflecting an attack rate of 5.8%. Sex ratio was 1.08. The patients were most often farmers (35.2%) and traders (33.3%). The working population (15-49 years old) accounted for 51.9%. The number of lesions varied between 1 and 5 in 91.7% of the cases. The lesions manifested as raised and infiltrated ulcerative lesions on the limbs (87%) with evolution ranging from 1 to 5 months in 96.3% of the cases. Samples were collected from two patients; microscopy showed leishmanias and PCR confirmed Leishmania major.

**Conclusion:**

our results confirm the presence of a cutaneous leishmaniasis major outbreak in the western part of the country. Additional surveys are needed to clarify the burden of leishmaniasis in Burkina Faso.

## Introduction

Les leishmanioses sont des affections parasitaires dues aux leishmanies et transmises à l’homme par la piqure de phlébotomes. Il s’agit d’un groupe de maladies extrêmement divers tant sur le plan clinique qu’épidémiologique. Ainsi on distingue: la leishmaniose viscérale (fièvre de Dumdum ou le Kala-azar); la leishmaniose cutanéo-muqueuse (Espundia ou Pian-bois ou uta) et la leishmaniose cutanée pure (Bouton d’Orient, Ouaga 2000, maladie de 6 mois…). Selon l’OMS, le poids des leishmanioses reste considérable [1]. Les leishmanioses sont des maladies tropicales négligées touchant principalement les pays en développement dont le Burkina Faso.

Au Burkina Faso, la leishmaniose cutanée (LC) à *Leishmania major* a été signalée suivant un mode endémique. Le premier cas date de 1960 [2] et quelques cas furent signalés dans les années suivantes. Mais cette maladie a connu une explosion épidémique en 1996 à Ouagadougou dans la région sanitaire du centre [3] (sous le nom de « maladie de ouaga 2000 » avec en moyenne plus de 1500 cas recensés dans les formations sanitaires de 2000 à 2005 [4]. Les facteurs expliquant les épidémies de LC à Ouagadougou sont toujours au stade d’hypothèses. En effet, l’espèce de phlébotome vectrice n’a toujours pas été mise en évidence [5]. Le rôle des déplacements des populations urbaines vers les zones périphériques a également été incriminé dans l’épidémie [4]. Les rongeurs du genre *Mastomys sp, Taterillus sp* ont été capturés infectés, suggérant leur rôle de réservoir de parasites dans l’épidémiologie.

Plusieurs études épidémiologiques, cliniques et biologiques ont concerné la leishmaniose cutanée dans cette région sanitaire du centre [4, 6, 7]. L’espèce retrouvée était *L. major* [8] et l’infection à VIH est ressortie comme facteur aggravant [9, 10, 11]. En dehors de cette région, la leishmaniose cutanée est notifiée dans toutes les autres régions sanitaires du pays sans confirmation biologique des cas [12]. Jusqu’à présent, le seul foyer confirmé au laboratoire et qui a fait l’objet d’études publiées à notre connaissance est celui de Ouagadougou. Dans l’ouest du Burkina Faso, la leishmaniose est peu décrite. Des cas isolés de suspicion de LC sont rencontrés en consultation de dermatologie au Centre Hospitalier Universitaire Souro Sanou (CHUSS) de Bobo-Dioulasso sans confirmation parasitologique. A travers la présente étude, nous rapportons les résultats épidémiologiques, cliniques et biologiques de l’investigation d’un foyer épidémique à Larama, district sanitaire de Karangasso Vigué dans la région des Hauts-Bassins à l’ouest du Burkina Faso.

## Méthodes

**Type et période d’étude:** l’étude s’est déroulée en 2 volets conjoints en septembre 2013. Un volet quantitatif qui a consisté à étudier les cas de leishmaniose à travers une enquête transversale descriptive. Ce volet a été complété par une étude qualitative basée sur des interviews de personnes clés et d’une inspection du cadre de vie.

**Cadre de l’étude:** l’étude s’est déroulée dans le village de Larama. Il est dans l’aire sanitaire du Centre de Santé et de Promotion Sociale (CSPS) de Soumousso et situé à environ 40km au sud-est de Bobo-Dioulasso. Sa population est estimée à 1852 habitants [13]. Le CSPS de Soumousso relève du district de Karangasso-Vigué dans la région sanitaire des Hauts Bassins. Le district de Karangasso-Vigué est logé dans le bassin de la Bougouriba. La pluviométrie moyenne annuelle est de 1143mm. La végétation est de type savane arbustive avec des zones arborées et des galeries forestières.

**Population d’étude et échantillonnage:** l’étude a concerné les habitants de Larama. Les patients ont été regroupés sur un site au centre de Larama choisi par la population elle-même pour l’inclusion. Tous les habitants présentant des lésions suspectes ont été invités pour participer à l’étude. Les personnes clés interviewés étaient des habitants de Larama.

**Critères d’inclusion:** tous les patients présentant des lésions répondant à la définition opérationnelle de cas suspect de leishmaniose ont été inclus dans l’étude quantitative. Les personnes interrogées dans l’étude qualitative ont été choisies de façon raisonnée.

**Critère de non inclusion:** les personnes qui ont refusé de participer n’ont pas été incluses.

**Variables étudiées:** les variables étudiées étaient quantitatives et qualitatives. Les variables quantitatives étaient en rapport avec les caractéristiques sociodémographiques (l’âge, le sexe, l’occupation principale), cliniques (durée d’évolution de la maladie, nombre de lésion, la localisation des lésions et le type de lésion) et biologiques (la microscopie, l’immunologie et la biologie moléculaire). Les variables qualitatives étaient en rapport avec l’environnement et les connaissances des populations sur la maladie.

**Définitions opérationnelles des cas:** pour décrire les cas, nous nous sommes inspirés du guide de surveillance intégrée de la maladie et la riposte du Ministère de la Santé du Burkina Faso [14]. Le cas suspect est toute personne présentant sur les parties découvertes (visage, cou, bras, jambes) un ou plusieurs nodule(s) et/ou une ou plusieurs ulcération(s) crouteuse(s) indolore(s) ne cicatrisant pas sous les traitements usuels antiseptique et antibiotique. Le cas confirmé est un cas suspect avec découverte des leishmanies dans les lésions.

**Technique de collecte des données:** les données ont été collectées par une équipe pluridisciplinaire faite de médecins spécialistes en santé publique, dermatologie, parasitologie, infectiologie, d’infirmiers et de logisticiens. La méthode du camp a été utilisée pour recevoir les cas [15]. Avant la réalisation du camp, la population a été informée par les relais communautaires sur la date et le lieu du camp. Les habitants présentant des lésions suspectes ont été invités à s’y présenter pour bénéficier d’un examen gratuit. Les données ont été recueillies à l’aide d’un questionnaire. Les cas suspects présents le jour du camp ont bénéficié d’un examen clinique pour leur inclusion éventuelle. Pour les examens complémentaires, nous avons fait un raclage des bords des lésions pour recueillir les sérosités sanguinolentes, une biopsie cutanée et un prélèvement de sang. Les sérosités sanguinolentes ont permis de réaliser un frottis pour l’observation microscopique après coloration au May Gunwald Giemsa (MGG). La biopsie cutanée à cheval sur les lésions après apposition sur lame a fait l’objet d’un examen microscopique. Une partie a été broyée pour l’extraction de l’ADN grâce au kit QUIAGEN. L’amplification à la PCR diagnostic a été faite selon le protocole de Noyes *et al*. 1998 [16]. Le prélèvement de sang veineux sur EDTA a été centrifugé à 3000 tours/min pendant 5min et le sérum a été testé avec le kit d’immunochromatographie (DiaMed-IT LEISH, Cressier sur Morat, Switzerland). Les données qualitatives ont été collectées à travers des interviews et une visite avec inspection du cadre de vie.

**Traitement des données:** les données ont été saisies sur Epi data et analysées avec Stata. Le taux d’attaque a été déterminé par le rapport (nombre de cas/population totale exposée). Des analyses uni et bivariées ont été faites pour la description des différentes variables.

**Aspects éthiques:** l’étude a été conduite sous l’égide du Ministère de la Santé (DLM, DRS). Les participants ont étés inclus après leur consentement verbal et les données compilées sont restées anonymes. Les participants ont bénéficié d’un examen clinique, d’examens complémentaires (pour certains), de conseil et de prescription gratuits.

## Résultats

**Caractéristiques épidémiologiques**

**Taux d’attaque:** au total, 108 cas suspects de leishmaniose ont été recensés. Tous les cas provenaient du village de Larama. Pour une population de 1852 habitants, le taux d’attaque est de 5,8%.

**Caractéristiques sociodémographiques:** le sex ratio était de 1,08 (hommes/femmes). La population active (15 à 49 ans) représentait 51,9%. Les cas étaient le plus souvent cultivateurs (35,2%) et commerçants (33,3%). Le Tableau 1 montre la répartition des cas suspects de leishmaniose selon l’âge, le sexe et l’occupation principale.

**Tableau 1:** Répartition des cas de leishmaniose cutanée selon les classes d’âges, le sexe, la profession et la durée d’évolution de la maladie

Caractéristiques	Nombre de cas	Proportions (%)
**Classes d’âges**		
Moins d’1 an	1	0,9
1 - 4 ans	16	14,8
5 - 14 ans	25	23,2
15 - 24 ans	24	22,2
25 - 49 ans	32	29,6
50 et plus	10	9,3
**Total**	108	100,0
**Sexe**		
Féminin	52	48,1
Masculin	56	51,9
**Total**	108	100,0
**Profession**		
Commerçants	36	33,3
Cultivateurs	38	35,2
Elève	8	7,4
Ménagère	22	20,4
Autres*	4	3,7
**Total**	108	100,0
**Durée d’évolution de la maladie**		
Moins d’un mois	4	3,7
2 à 3 mois	53	49,1
4 à 5 mois	51	47,2
**Total**	108	100,0

(*) = les autres professions étaient 1 tailleur, 1 marabout et 2 éleveurs.

### Caractéristiques cliniques

**Figure 1:**
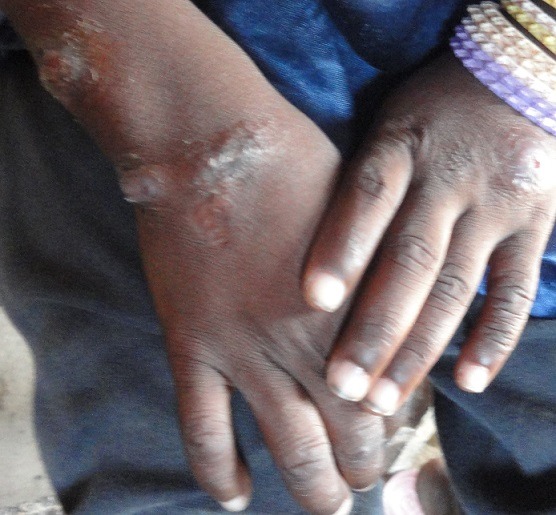
Forme ulcéro-croûteuse, surélevée et infiltrée de leishmaniose cutanée des extrémités

**Figure 2:**
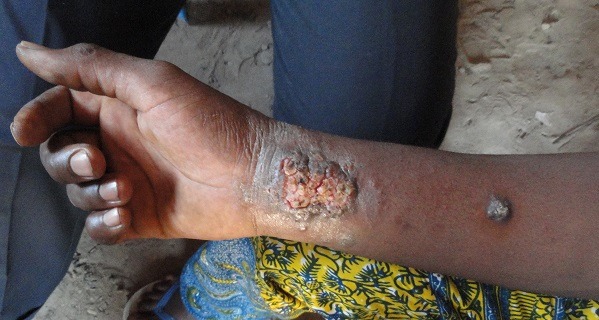
Forme ulcéro-croûteuse, surélevée et infiltrée de leishmaniose cutanée du poignet

**Figure 3:**
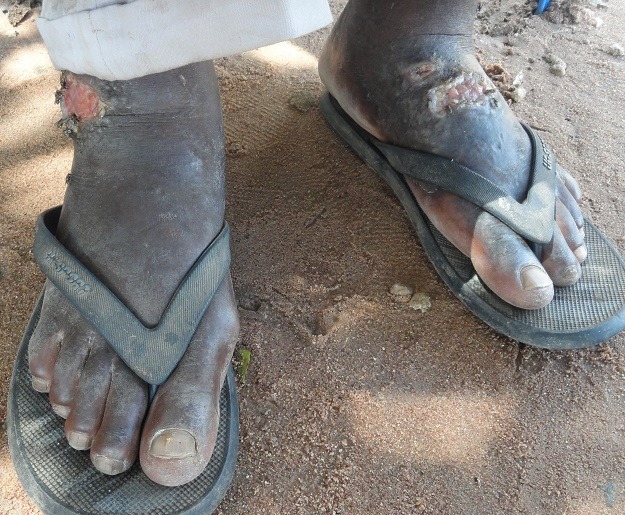
Forme ulcéro-croûteuse, surélevée et infiltrée de leishmaniose cutanée des pieds

**Figure 4:**
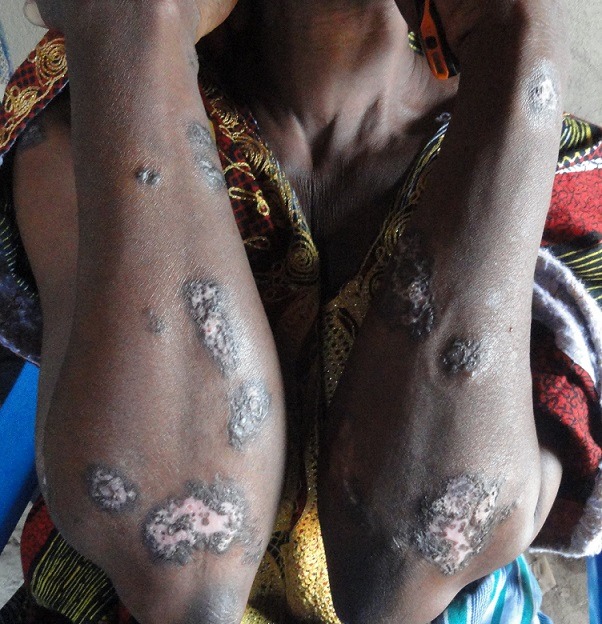
Lésions cicatricielles de leishmaniose cutanée des avant-bras

Selon la population, la maladie évoluait depuis environ 6 mois. Le Tableau 1 montre la répartition des cas suspects selon la durée d’évolution. Le nombre de lésions s’étend de 1 à 40 par malade avec une moyenne de 5,6 lésions par malades. Les sièges les plus fréquents des lésions se situent au niveau des membres (87%) (Figure 1, Figure 2, Figure 3 et Figure 4). Les autres localisations étaient le dos, le thorax, le visage, la tête et l’abdomen. Chez 5,6% des cas, les lésions étaient généralisées à tout le corps. Les lésions observées avaient un aspect ulcéro-crouteux, surélevé et infiltré (Figure 1, Figure 2 et Figure 3); d’évolution chronique avec un aspect cicatriciel par endroit (Figure 4).

### Caractéristiques biologiques

Sur le plan biologique, nous avons choisi de prélevé 2 cas suspects. L’immunologie était franchement positive sur un prélèvement et négative sur l’autre. La microscopie a montré au MGG la présence d’amastigotes de leishmanies chez les deux patients et à la PCR, les 2 cas étaient positifs à *Leishmania major*.

### Les données qualitatives

La synthèse de ces données a montré les éléments pertinents suivants: 1) La maladie n’était pas connue des habitants du village. Un homme âgé d’environ 80 ans, résident permanent du village depuis sa naissance atteste que c’est la première fois qu’il voit cette maladie; 2) La maladie évolue depuis environ 6 mois dans le village; 3) La maladie a débuté chez un homme de 62 ans environ qui se reconnait comme cas index. Il a séjourné dans le district de Mangodara (district voisin) 2 mois avant le début de l’apparition de ses lésions; 4) Il existe un grand marabout dans le village qui est fréquenté par plusieurs personnes venant des horizons divers; 5) Le village dispose d’un marché fréquenté par les populations environnantes et les habitants du village fréquentent les villages voisins pour faire le marché ou participer à des cérémonies (mariage, funérailles…); 6) Deux villages voisins abritent des sites aurifères; 7) Il n’a pas été retrouvé de bouleversement écologique, de migration/émigration, de pullulation de canidés majeurs. Cependant, le village se trouve dans une zone très arborée et humide. Les conditions d’hygiène et d’assainissement sont précaires. Quelques animaux domestiques cohabitent avec les habitants.

## Discussion

Notre étude a utilisé la double approche quantitative et qualitative pour décrire un nouveau foyer de leishmaniose cutanée. La méthode du camp utilisée a été validée par le groupe d’expert de l’OMS dans le cadre de la lutte contre la leishmaniose [15]. La confirmation biologique n’a pas été systématique compte tenu du caractère invasif des prélèvements et de l’évidence clinique des lésions. L’étude n’a pas pu être accompagnée d’enquête entomologique et d’une étude sur les réservoirs pour des raisons budgétaires. Néanmoins, elle a permis à l’aide d’une équipe pluridisciplinaire de confirmer et de donner des informations pertinentes sur un nouveau foyer épidémique de leishmaniose cutanée et probablement le premier foyer dans l’ouest du Burkina Faso.

### Des caractéristiques épidémiologiques

Elles ont étés collectées par la méthode du camp. Cette méthode consiste à regrouper les cas suspects sur un site facile d’accès et de commun accord avec la population. Elle est d’autant plus fiable que la maladie est facilement reconnaissable, comme cela l’est dans la leishmaniose cutanée. Elle a l’insuffisance de ne pas prendre en compte les cas qui ne vont pas se déplacer au camp pour diverses raisons.

**Du taux d’attaque:** le taux est de 5,8%. Il s’agit ici d’un taux probablement sous-estimé du fait de la méthode du camp qui a été utilisée. En effet, certains cas de leishmaniose pourraient ne pas se rendre au camp pour diverses raisons comme le manque d’information, l’atypie des lésions, les conflits d’agenda avec les autres activités champêtres, commerciales ou socio-culturelles ou tout simplement par manque de motivation. Cette sous-estimation est certainement minime car Larama est un petit village où les habitants se connaissent. De plus, la population a participé à l’organisation du camp et au choix du site. La recherche des cas par la méthode du porte en porte aurait minimisé ces éléments mais sa réalisation serait difficile en cette période hivernale de septembre pendant laquelle l’accès aux habitations n’est pas toujours aisé [15].

**Des caractéristiques sociodémographiques:** les tranches d’âges actives adultes étaient les plus touchées. Cela est habituel dans les zones qui n’ont pas connu de cas de leishmaniose avant l’épidémie et Larama semble n’avoir pas connu de leishmaniose auparavant selon la population. En effet, dans ces zones, les adultes sont aussi atteints et parfois même plus atteints que les enfants car le manque d’immunité anti leishmanienne concerne l’ensemble de la population sans distinction d’âge. Dans les zones d’endémie leishmanienne, les adultes sont plutôt moins touchés par la maladie du fait de leur immunité comme c’est le cas dans l’étude de Ngouateu *et al*. au Cameroun [17]. Ce résultat vient renforcer le fait que Larama soit un nouveau foyer de leishmaniose cutanée. Les cultivateurs, les commerçants ainsi que les ménagères semblent les plus atteints. En effet, ils sont plausiblement les plus exposés aux piqûres de phlébotomes car leurs occupations les poussent à rester hors des domiciles pour ne rentrer que les soirs.

### Des caractéristiques cliniques

Pour la plupart des cas, les lésions ont débuté entre les mois de mars et de mai. Cela semble habituel pour la leishmaniose au Burkina Faso [3, 4, 6]. Ces mois correspondent à la période chaude de l’année. Les populations ont tendance à ne pas porter de vêtement et à dormir en dehors des maisons. Cette période correspond aussi au début des travaux champêtres qui obligent la population active à sortir des maisons. Les lésions étaient de type ulcéro-crouteux comme ce qui s’observe dans les épidémies à *Leishmania major* [7]. Elles étaient localisées sur les parties découvertes, excepté le visage qui était peu atteint. Ces parties du corps sont celles exposées aux piqures de phlébotome et les lésions de leishmaniose cutanée (LC) se développent sur les sites de piqûre. La sensibilité du visage augmente la vigilance de l’humain sur cette partie et la rend relativement moins accessible aux phlébotomes.

### Des caractéristiques biologiques

Nous avons choisi de prélever deux patients pour la confirmation biologique. L’idéale serait d’avoir un prélèvement pour tous les cas. Le caractère invasif des prélèvements et le nombre de tests de laboratoires limités nous ont imposé cette limitation. De plus, la leishmaniose est une affection bien connue des dermatologues burkinabès à travers le foyer de Ouagadougou. Les examens biologiques, à savoir la microscopie et la biologie moléculaire confirment qu’ils s’agissaient bien de leishmanioses cutanées et avec *L. major* comme espèce incriminée. Ce résultat est en accord avec la clinique (lésion ulcéreuse humide due aux espèces zoonotiques de leishmanies telles que *L. major* [7] et avec la littérature sur la leishmaniose au Burkina Faso. En effet, l’espèce notifiée au Burkina Faso à l’origine de la leishmaniose cutanée est *L. major* MON-74 [8]. Nous avons eu un échantillon positif sur deux à l’immunologie. La positivité de ce test est étonnante car élaboré pour *L. infantum* pour les anticorps IgM/IgG (espèce de leishmanie viscéralisante) avec une spécificité et sensibilité supérieure à 95%. Même si une étude récente a notifié la circulation de *L. infantum* chez les chiens domestiques [18], nous expliquons la positivité du test immunologique par les possibles réactions croisées entre espèces leishmaniennes du fait de l’homologie antigénique [19].

### Des données qualitatives

L’enquête qualitative a permis de donner des éléments de précision par rapport à l’épidémie. En effet, les échanges directs avec les populations ont permis de noter qu’il s’agissait des premiers cas du village avec l’identification du cas index. Le séjour de ce cas index en dehors de Larama avant le début de la maladie ouvre la discussion sur l’existence de cas sur son itinéraire. Il pourrait aussi s’agir de cas importé autrement, compte tenu des multiples mouvements de population liés aux cérémonies, aux marchés, aux sites aurifères ou même aux clients du marabout basé à Larama. Ces éléments sont confortés par le manque de bouleversement écologique récent lié aux déplacements de population. La cohabitation des animaux domestiques, des animaux sauvages avec pour corolaire l’insalubrité des habitations dans un environnement arboré serait des éléments qui ont pu contribuer à l’expansion de l’épidémie. En effet, les rongeurs sont les principaux réservoirs décrits de *L. major*. L’enquête entomologique et l’étude des réservoirs sont des perspectives intéressantes pour décrire l’épidémiologie de cette parasitose émergente.

## Conclusion

La leishmaniose cutanée à *Leishmania major* est bien connue au Burkina Faso. Elle semblait être l’apanage du centre du pays et probablement du nord. La zone ouest semblait jusqu’à présent peu touchée. Avec les mouvements de population, il n’est pas étonnant qu’une épidémie se retrouve loin des foyers habituels. Cela nous amène à nous interroger sur l’existence de la LC dans les autres parties du pays. Des enquêtes complémentaires sont nécessaires pour préciser la distribution des vecteurs et la charge de morbidité de cette affection au Burkina.

### Etat des connaissances actuelles sur le sujet

- La leishmaniose cutanée est endémique au Burkina Faso avec un foyer épidémique au centre.

### Contribution de notre étude à la connaissance

- L’étude a permis de mettre en évidence un nouveau foyer épidémique à *Leishmania major* dans une région qui ne connaissait pas de leishmaniose;
- Elle permet ainsi d’interpeller sur la recherche active des cas à travers le Burkina Faso.

## Conflits d’intérêts

Les auteurs ne déclarent aucun conflit d’intérêts.
